# The causality between gut microbiota and non-Hodgkin lymphoma: a two-sample bidirectional Mendelian randomization study

**DOI:** 10.3389/fmicb.2024.1403825

**Published:** 2024-05-27

**Authors:** Jinjie Fu, Zheng Hao

**Affiliations:** ^1^Graduate School, Tianjin University of Traditional Chinese Medicine, Tianjin, China; ^2^College of Traditional Chinese Medicine, Tianjin University of Traditional Chinese Medicine, Tianjin, China; ^3^Tianjin Key Laboratory of Modern Chinese Medicine Theory of Innovation and Application, Tianjin, China; ^4^Guo Aichun Institute of Medical History and Literature, Tianjin University of Traditional Chinese Medicine, Tianjin, China

**Keywords:** gut microbiota, non-Hodgkin lymphoma, diffuse large B-cell lymphoma, haematology system, Mendelian randomization

## Abstract

**Background:**

Studies have indicated an association between gut microbiota (GM) and non-Hodgkin lymphoma (NHL). However, the causality between GM and NHL remains unclear. This study aims to investigate the causality between GM and NHL using Mendelian randomization (MR).

**Methods:**

Data on GM is sourced from the MiBioGen consortium, while data on NHL and its subtypes is sourced from the FinnGen consortium R10 version. Inverse variance weighted (IVW) was employed for the primary MR analysis method, with methods such as Bayesian weighted Mendelian randomisation (BWMR) as an adjunct. Sensitivity analyses were conducted using Cochran’s Q test, MR-Egger regression, MR-PRESSO, and the “Leave-one-out” method.

**Results:**

The MR results showed that there is a causality between 27 GMs and NHL. Among them, 20 were negatively associated (OR < 1), and 7 were positively associated (OR > 1) with the corresponding diseases. All 27 MR results passed sensitivity tests, and there was no reverse causal association.

**Conclusion:**

By demonstrating a causal link between GM and NHL, this research offers novel ideas to prevent, monitor, and cure NHL later.

## 1 Introduction

NHL is a prevalent malignancy tumor in the haematology system, accounting for about 90% of lymphomas overall. It can be classified into three basic types: B-cell type, T-cell type, and NK-cell type (Shankland et al., 2012). The prevalence of NHL is progressively rising on an annual basis. Based on statistical data, the number of new NHL cases in 2020 was 544,000, with approximately 260,000 deaths (Mafra et al., 2022). The number of new cases is projected to reach 778,000 by 2040, an increase of about 43% compared to 2020 (Chu et al., 2023). While the etiology of NHL is not fully understood, infection, immunosuppression, immunodeficiency syndromes, and autoimmune diseases are commonly recognized as significant risk factors for the onset of NHL (Ansell, 2015; Armitage et al., 2017). In terms of treatment, from the anti-CD20 monoclonal antibody (rituximab) in 1982 (Miller et al., 1982), to the current immune checkpoint inhibitors (ICI) and bispecific antibodies (Bock et al., 2022; Abou Dalle et al., 2024), immunotherapy combined with chemotherapy has always been a focus in the treatment of NHL. Despite some progress made in these treatment methods, the therapy of relapsed/refractory NHL is still a major dilemma in the field, with many unmet needs in NHL therapy (Chaudhari et al., 2019).

The gastrointestinal tract, as the most common extranodal site involved in NHL (Hanafy et al., 2020), harbors a large number of microbes, such as bacteria and fungi. This subset of microorganisms is collectively referred to as GM (Costea et al., 2017). Recently, the close connection between GM and NHL has been increasingly confirmed. Research has shown that the abundance of GM in diffuse large B-cell lymphoma (DLBCL) patients is markedly greater than that in healthy individuals, as revealed by 16S rRNA gene sequencing (Yuan et al., 2021). In terms of NHL occurrence, studies have found an association between mucosa-associated lymphoid tissue (MALT) lymphoma and the invasion of GM such as Burkholderia. GM like Burkholderia may influence the mechanism of MALT lymphoma occurrence through the synthesis of Mvin protein (Kuo et al., 2019; Tanaka et al., 2021). Regarding the diagnosis of NHL, some scholars have suggested that GM can serve as a diagnostic marker for NK/T cell lymphoma (Shi et al., 2023). In addition, GM can also modulate the efficacy of immunotherapy. Studies have shown that the treatment response of cancer patients receiving immune checkpoint inhibitors (ICIs) is associated with the composition of GM. For example, GM such as Bacteroides may enhance patients’ anti-tumor capacity by improving the function of effector T cells in the tumor microenvironment (Gopalakrishnan et al., 2018). Furthermore, studies have shown that oral administration of *Akkermansia muciniphila* and fecal microbiota transplantation can restore the efficacy of immune checkpoint inhibitors (ICI) in drug-resistant tumor mice through an interleukin-12-dependent mechanism (Routy et al., 2018). Therefore, by modulating GM, it is possible to improve the therapeutic effect of immunotherapies such as ICB, lower associated side effects (Park E. M. et al., 2022), and mitigate the development of resistance to ICIs in cancer patients (Routy et al., 2018). Myeloablative conditioning and the use of broad-spectrum antibiotics before hematopoietic stem cell transplantation (HSCT) can damage the intestinal epithelium and mucosal barrier, leading to gastrointestinal mucositis, and consequently increasing the risk of infections in patients (Keefe et al., 2007). Meanwhile, GM can influence the immune system and maintain intestinal homeostasis by regulating cells such as Treg and TH17 (Arpaia et al., 2013; Smith et al., 2013). Based on differences in GM, it is possible to predict and assess pre-transplant risks in NHL patients undergoing HSCT, aiding in the identification and prevention of high-risk individuals (Montassier et al., 2016). For instance, assessing the diversity of gut microbiota (GM) in patients on the day of transplant surgery can predict those at high risk of mortality during HSCT (Taur et al., 2014). In the future, GM may be a novel diagnostic biomarker and therapeutic target for NHL. Therefore, research on the causal relationship between the two is necessary.

MR explores the causality between exposure and outcome by utilizing instrumental variables (IVs) (Davies et al., 2018). Under the principle of random assignment, MR studies could avoid confounding factors or reverse causation interference (Davey Smith and Hemani, 2014), resulting in more stable and reliable research outcomes. For the research, we employ a two-sample MR methodology to investigate the causality between GM and NHL.

## 2 Methods

### 2.1 Data sources

MiBioGen consortium provided genetic variation data on GM (Kurilshikov et al., 2021). This research involved 18, 340 persons and generated corresponding genetic sequencing and genotyping data. It included 211 GMs, classified into five categories: phylum, class, order, family, and genus. Three unknown families and twelve unknown genera were excluded. Eventually, the study included nine phyla, sixteen classes, twenty orders, thirty-two families, and one hundred nineteen genera, totaling 196 GMs. The genetic variation data for NHL originates from the FinnGen consortium R10 version GWAS summary data (Kurki et al., 2023). It includes NHL and its five subtypes: follicular lymphoma (FL), DLBCL, marginal zone B-cell lymphoma (MZBL), T/NK cell lymphoma, and mantle cell lymphoma (MCL) (Table 1). The diagnostic criteria for NHL refer to ICD-10 codes C82, C83, C84, C85; The diagnostic criteria for FL refer to ICD-10 code C82; The diagnostic criteria for DLBCL refer to ICD-10 code C83.3; The diagnostic criteria for MZBL refer to ICD-10 codes C83.80, C83.89; The diagnostic criteria for T/NK cell lymphoma refer to ICD-10 code C84; The diagnostic criteria for MCL refer to ICD-10 code C83.1.

**Table 1:** Detailed information on GMs and NHLs with their subtypes.

	Trait	Year	Population	Case	Control	PMID/URL (Datadownload)
Exposure	Gut microbiota	2023	European	–	–	33462485
Outcome	Non-Hodgkin lymphoma	2023	European	1,072	314193	https://storage.googleapis.com/finngen-public-data-r10/summary_stats/finngen_R10_C3_NONHODGKIN_EXALLC.gz
	Follicular lymphoma	2023	European	1,181	324650	https://storage.googleapis.com/finngen-public-data-r10/summary_stats/finngen_R10_CD2_FOLLICULAR_LYMPHOMA_EXALLC.gz
	Diffuse large B-cell lymphoma	2023	European	1,050	314193	https://storage.googleapis.com/finngen-public-data-r10/summary_stats/finngen_R10_C3_DLBCL_EXALLC.gz
	Marginal zone B-cell lymphoma	2023	European	202	314193	https://storage.googleapis.com/finngen-public-data-r10/summary_stats/finngen_R10_C3_MARGINAL_ZONE_LYMPHOMA_EXALLC.gz
	Mantle cell lymphoma	2023	European	210	314193	https://storage.googleapis.com/finngen-public-data-r10/summary_stats/finngen_R10_C3_MANTLE_CELL_LYMPHOMA_EXALLC.gz
	T/NK-cell lymphomas	2023	European	363	324650	https://storage.googleapis.com/finngen-public-data-r10/summary_stats/finngen_R10_CD2_TNK_LYMPHOMA_EXALLC.gz

### 2.2 Selection of IVs

**Figure 1:**
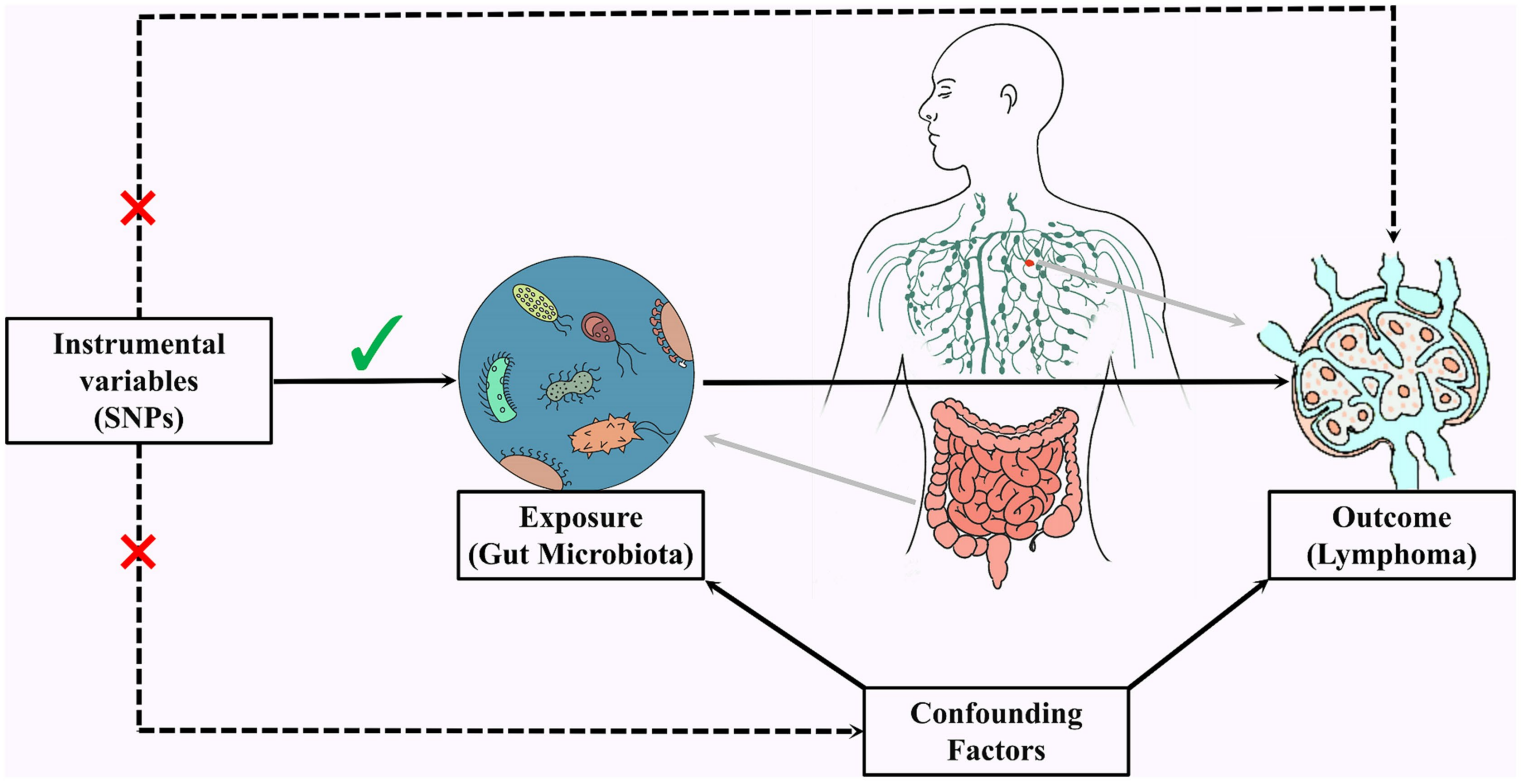
Three conditions met by filtering IVs.

IVs were screened based on the following criteria (Figure 1): (1) In order to obtain IVs that are strongly correlated with GM and have robust relationships, we set the significance threshold at *p* < 1.0 × 10^−8^. However, the number of obtained IVs was small and difficult to meet the requirements of this study. Therefore, we referred to previous research (Sanna et al., 2019) and set the significance threshold at *p* < 1.0 × 10^−5^. At the same time, to ensure the mutual independence of the selected IVs, we removed linkage disequilibrium in the IVs (*r*^2^ < 0.001, kb = 10,000); (2) To ensure the independence of the IVs, the IVs are unrelated to any confounding factors; (3) IVs can only influence the occurrence of NHL through GM and cannot affect NHL through other pathways. Additionally, to avoid bias from weak IVs, we excluded weak IVs using the F-statistic (*F* > 10). We also removed palindromic sequences from the IVs.

### 2.3 Positive MR analysis and sensitivity analysis

We utilized six methods, including IVW, MR Egger, weighted median, simple mode, weighted mode, and BWMR, to investigate the causality between GM and NHL. However, IVW was the primary evaluation method (Hemani et al., 2018). Given the uniqueness of GWAS data, the IVW method is widely recognized as the primary method for exploring causal relationships in MR analysis. By conducting a meta-analysis of each Wald ratio of the included valid IVs, it can provide the most accurate estimate of the effect. This approach is also commonly seen in other literature (Legason et al., 2017; Lu et al., 2023; Martín-Masot et al., 2023; Ruan et al., 2023; Li et al., 2024; Zheng et al., 2024). The IVW method is divided into random-effects IVW and fixed-effects IVW, with the selection based on heterogeneity in MR results (Greco et al., 2015). Since no single method can perfectly suit all situations, additional methods such as MR Egger and weighted median are used as supplements (Bowden et al., 2015, 2016). For instance, when there is pleiotropy present, the MR Egger method is more suitable for inferring causal relationships. Finally, to mitigate the effects of multi-genic structure and pleiotropy, we utilized the BWMR method to further validate the obtained causal relationships (Zhao et al., 2020).

Sensitivity analysis includes heterogeneity testing, leave-one-out testing, and multivariate testing (Hemani et al., 2018). We evaluated the potential bias in the results by examining the pleiotropy of genes and the heterogeneity of the data. Cochran’s Q test assesses heterogeneity, based on whether the *p*-value in Cochran’s IVW is less than 0.05. MR-Egger regression detects horizontal pleiotropy, determined by the difference between its intercept and 0. MR-PRESSO can detect and lower horizontal pleiotropy (Burgess et al., 2020). Additionally, “leave-one-out” analysis can identify outlier SNPs within the SNPs, thus avoiding bias introduced by individual outlier SNPs on the overall MR results and enhancing the stability of the results.

### 2.4 Reverse MR analysis

In order to avoid interference from reverse causal relationships on the positive MR results, we conducted a reverse MR analysis with NHL and its five subtypes as exposure and GM as the outcome.

### 2.5 Statistical analysis

The statistical analyses in R 4.3.0 used the “TwoSampleMR” package.

## 3 Results

### 3.1 Obtained IVs

412 IVs related to non-Hodgkin lymphoma and its subtypes were obtained through screening (Supplementary Table S1). Among them, there were no palindromic sequences, and the *F* > 10 (range 17.421–88.429). The included GMs were divided into five categories, so there may be overlaps among SNPs under each GM.

### 3.2 Results of positive MR analysis

**Figure 2:**
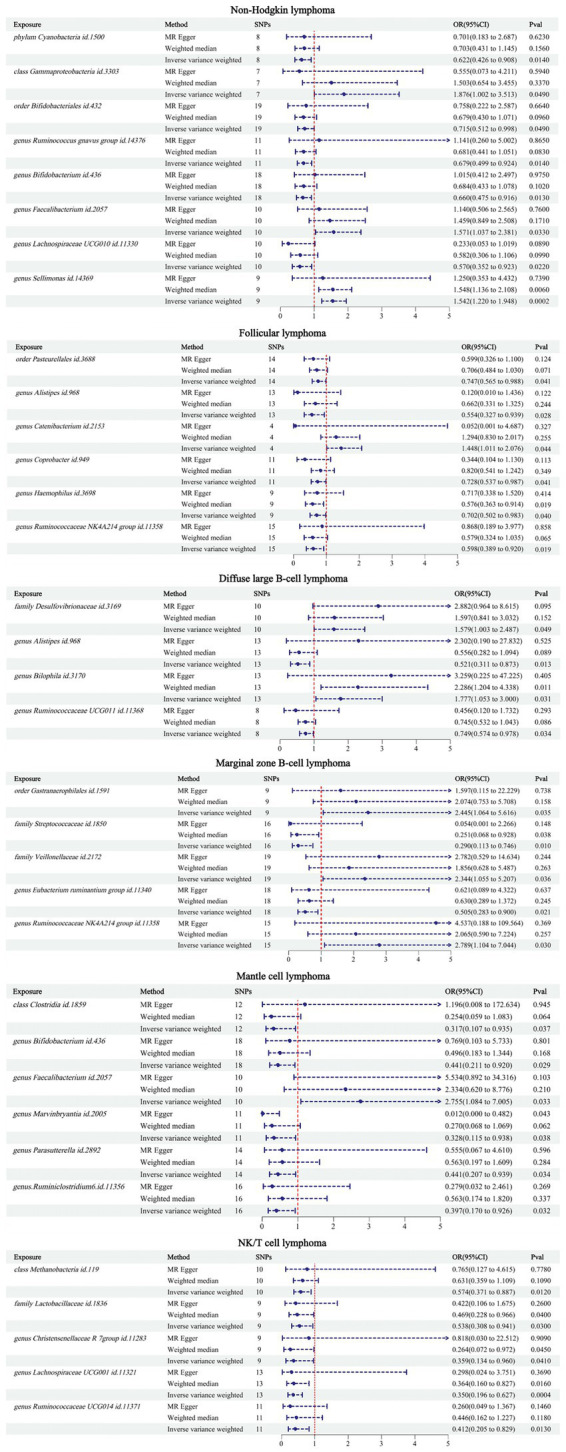
MR results between 34 GMs and NHL.

The IVW results showed that there were 34 GMs associated with NHL and its subtypes. Among them, 8 were associated with NHL, 6 with FL, 4 with DLBCL, 5 with MZBL, 6 with MCL, and 5 with T/NK cell lymphoma (Figure 2).

According to the IVW analysis results, phylum Cyanobacteria (OR: 0.622, CI: 0.426–0.908, *p* = 0.014), order Bifidobacteriales (OR: 0.715, CI: 0.512–0.998, *p* = 0.049), genus *Ruminococcus gnavus* group (OR: 0.679, CI: 0.499–0.924, *p* = 0.014), genus *Bifidobacterium* (OR: 0.660, CI: 0.475–0.916, *p* = 0.013), and genus Lachnospiraceae UCG010 (OR: 0.570, CI: 0.352–0.923, *p* = 0.022) were negatively associated with an increased risk of NHL. Class Gammaproteobacteria (OR: 1.876, CI: 1.002–3.513, *p* = 0.049), genus *Faecalibacterium* (OR: 1.571, CI: 1.037–2.381, *p* = 0.033), and genus *Sellimonas* (OR: 1.542, CI: 1.220–1.948, *p* = 0.0002) were positively associated with an increased risk of NHL.

According to the IVW analysis results, order Pasteurellales (OR: 0.747, CI: 0.565–0.988, *p* = 0.041), genus *Alistipes* (OR: 0.554, CI: 0.327–0.939, *p* = 0.028), genus *Coprobacter* (OR: 0.728, CI: 0.537–0.987, *p* = 0.041), genus *Haemophilus* (OR: 0.702, CI: 0.502–0.983, *p* = 0.040), and genus Ruminococcaceae NK4A214 group (OR: 0.598, CI: 0.389–0.920, *p* = 0.019) were negatively associated with an increased risk of FL. Genus *Catenibacterium* (OR: 1.448, CI: 1.011–2.076, *p* = 0.044) was positively associated with an increased risk of FL.

According to the IVW analysis results, genus *Alistipes* (OR: 0.521, CI: 0.311–0.873, *p* = 0.013), genus Ruminococcaceae UCG011 (OR: 0.749, CI: 0.574–0.978, *p* = 0.034) were negatively associated with an increased risk of DLBCL. Family Desulfovibrionaceae (OR: 1.579, CI: 1.033–2.487, *p* = 0.049), genus *Bilophila* (OR: 1.777, CI: 1.053–3.000, *p* = 0.031) were positively associated with an increased risk of DLBCL.

According to the IVW analysis results, family Streptococcaceae (OR: 0.290, CI: 0.113–0.746, *p* = 0.010), genus *Eubacterium ruminantium* group (OR: 0.505, CI: 0.283–0.900, *p* = 0.021) were negatively associated with an increased risk of MZBL. Order Gastranaerophilales (OR: 2.445, CI: 1.064–5.616, *p* = 0.035), family Veillonellaceae (OR: 2.344, CI: 1.055–5.207, *p* = 0.036), genus Ruminococcaceae NK4A214 group (OR: 2.789, CI: 1.104–7.044, *p* = 0.030) were positively associated with an increased risk of MZBL.

According to the IVW analysis results, class Clostridia (OR: 0.317, CI: 0.107–0.935, *p* = 0.037), genus *Bifidobacterium* (OR: 0.441, CI: 0.211–0.920, *p* = 0.029), genus *Marvinbryantia* (OR: 0.328, CI: 0.115–0.938, *p* = 0.038), genus *Parasutterella* (OR: 0.441, CI: 0.207–0.939, *p* = 0.034), genus *Ruminiclostridium* 6 (OR: 0.397, CI: 0.70–0.926, *p* = 0.032) were negatively associated with an increased risk of MCL. Genus *Faecalibacterium* (OR: 2.755, CI: 1.084–7.005, *p* = 0.033) was positively associated with an increased risk of MCL.

According to the IVW analysis results, class Methanobacteria (OR: 0.574, CI: 0.371–0.887, *p* = 0.012), family Lactobacillaceae (OR: 0.538, CI: 0.308–0.941, *p* = 0.030), genus Christensenellaceae R 7group (OR: 0.359, CI: 0.134–0.960, *p* = 0.041), genus Lachnospiraceae UCG001 (OR: 0.350, CI: 0.196–0.627, *p* = 0.0004), genus Ruminococcaceae UCG014 (OR: 0.412, CI: 0.205–0.829, *p* = 0.013) were negatively associated with an increased risk of T/NK cell lymphoma.

**Figure 3:**
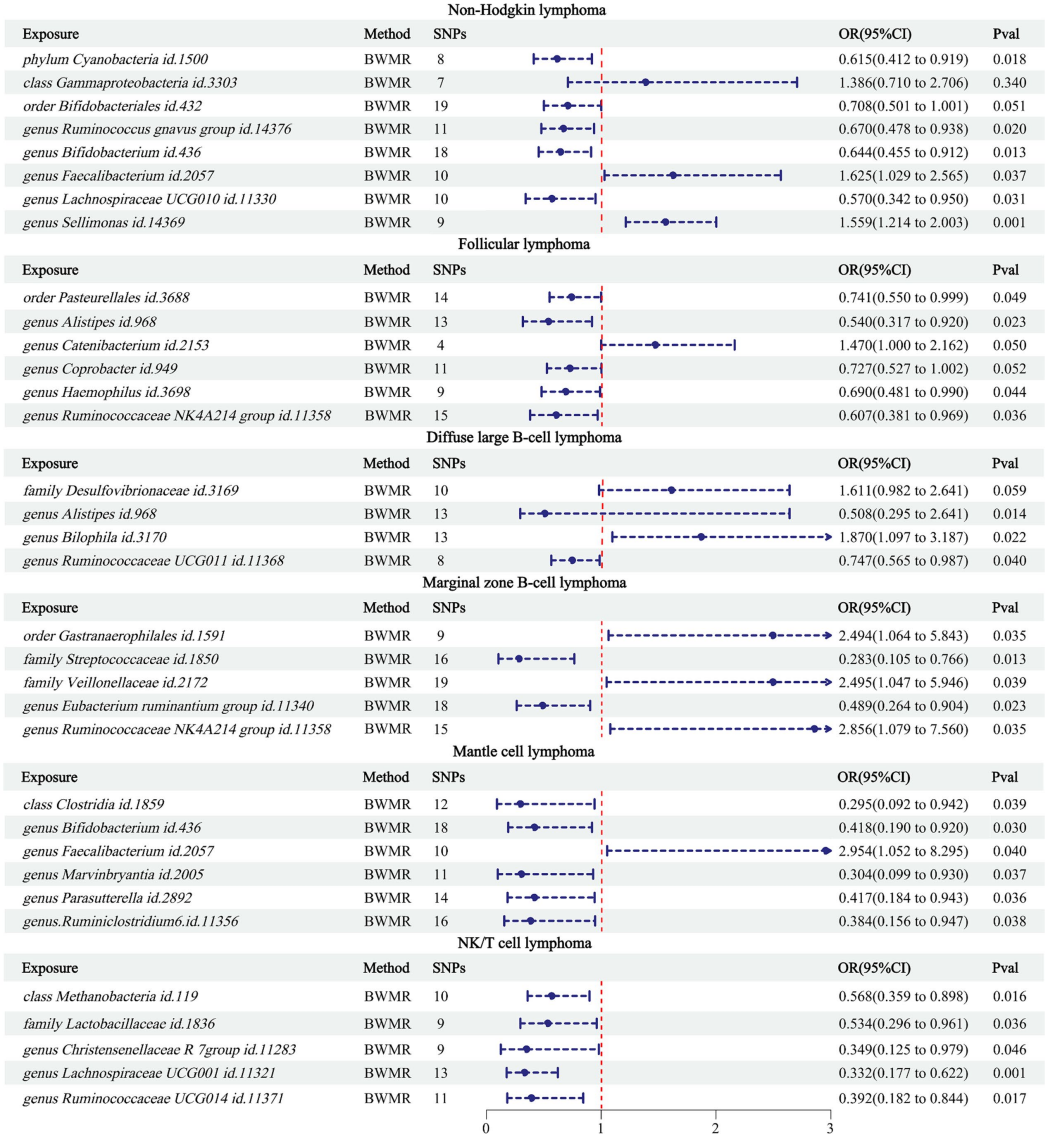
BWMR results between 34 GMs and NHL.

Using BWMR to validate the 34 pairs of causalities between the obtained GMs and NHL (Figure 3), the results showed that class Gammaproteobacteria (*p* = 0.340) and order Bifidobacteriales (*p* = 0.051) were not causally related to NHL; genus *Catenibacterium* (*p* = 0.050) and genus *Coprobacter* (*p* = 0.052) were not causally related to FL; family Desulfovibrionaceae was not causally related to DLBCL. Comparing these 5 pairs of relationships between the results of BWMR and IVW (Table 2), it was found that although causal relationships existed in the IVW results, their *p*-values were close to 0.05. Therefore, these 5 pairs of relationships were excluded from our study.

**Table 2:** Comparison of IVW and BWMR results for 5 pairs of GMs and NHL.

Exposure	Outcome	IVW		BWMR	
		Pval	OR(95%CI)	Pval	OR(95%CI)
*class Gammaproteobacteria id.3303*	Non-Hodgkin lymphoma	0.049	1.876 (1.002–3.513)	0.340	1.386 (0.710–2.706)
*order Bifidobacteriales id.432*		0.049	0.715 (0.512–0.998)	0.051	0.708 (0.501–1.001)
*genus Catenibacterium id.2153*	Follicular lymphoma	0.044	1.448 (1.011–2.076)	0.050	1.470 (1.000–2.162)
*genus Coprobacter id.949*		0.041	0.728 (0.537–0.987)	0.052	0.727 (0.527–1.002)
*family Desulfovibrionaceae id.3169*	Diffuse large B-cell lymphoma	0.049	1.579 (1.003–2.487)	0.059	1.611 (0.982–2.641)

Finally, sensitivity analysis of the MR results was conducted (Supplementary Table S1). The *p*-values of Cochran’s Q test were all >0.05, indicating no heterogeneity. The *p*-values of the MR-Egger intercept (Supplementary Figures S1, S2) and MR-PRESSO results were all >0.05, indicating no horizontal pleiotropy. Results from the “leave-one-out” method (Supplementary Figures S3, S4) showed that removing any single SNP would not significantly affect the MR results.

### 3.3 Results of reverse MR analysis

**Figure 4:**
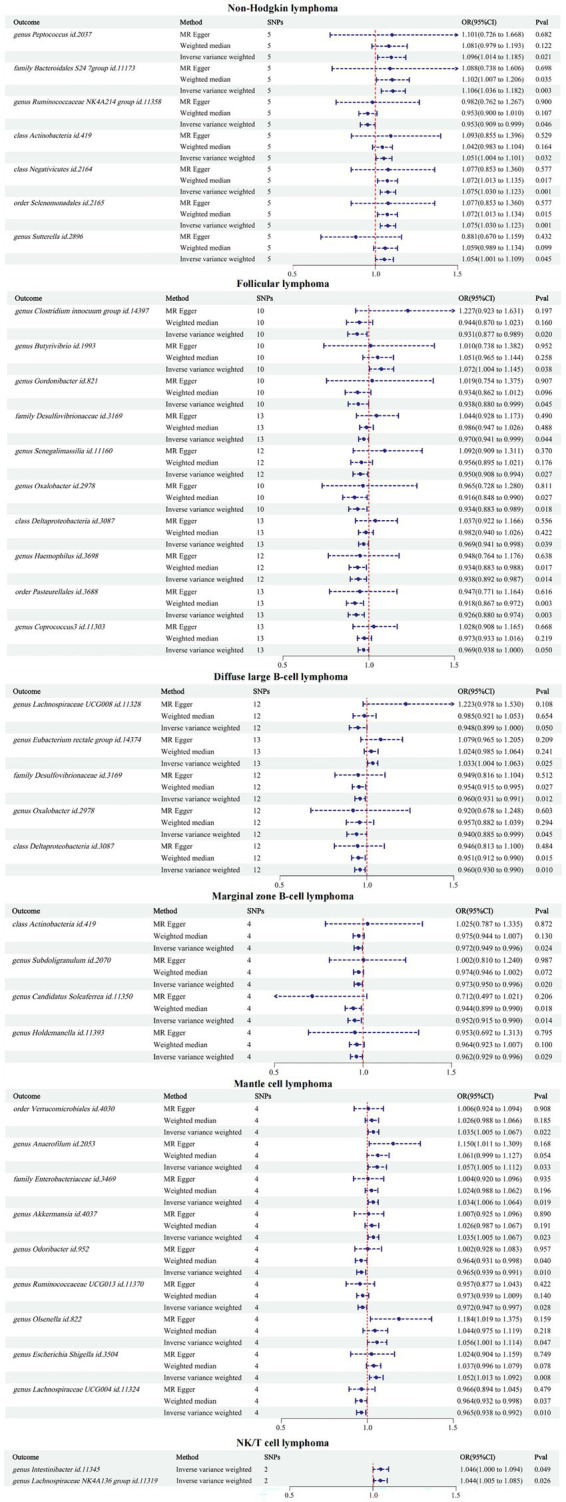
Inverse MR results between GMs and NHL.

The reverse MR results showed that NHL and its subtypes were associated with 37 GMs (Supplementary Table S3). Among them, there were 7 associated with NHL, 10 with FL, 5 with DLBCL, 4 with MZBL, 9 with MCL, and 2 with T/NK cell lymphoma. Mapping the forest, see Figure 4. After comparing with the results of the forward MR, among the 34 forward MR results, FL was inversely associated with order Pasteurellales and genus *Haemophilus*, DLBCL was inversely associated with family Desulfovibrionaceae, and no other reverse causal associations were found in the remaining forward MR results. Sensitivity analysis was conducted for the three reverse causal associations mentioned above (Table 3). Except for the presence of heterogeneity in the MR results between FL and order Pasteurellales (without horizontal pleiotropy), the remaining two MR results showed no heterogeneity or horizontal pleiotropy.

**Table 3:** Sensitivity analysis results for 3 GMs and NHL inverse MR results.

Exposure	Outcome	Q	Q_pval	MR-Egger intercept test		MR-PRESSO global test	
				Egger_intercept	pval	RSS obs	*p*-value
Follicular lymphoma	*order Pasteurellales*	21.424	0.045	−0.006	0.828	25.277	0.058
	*genus Haemophilus*	18.885	0.063	−0.003	0.925	22.189	0.087
Diffuse large B-cell lymphoma	*family Desulfovibrionaceae*	2.775	0.993	0.003	0.876	111.769	0.468

**Figure 5:**
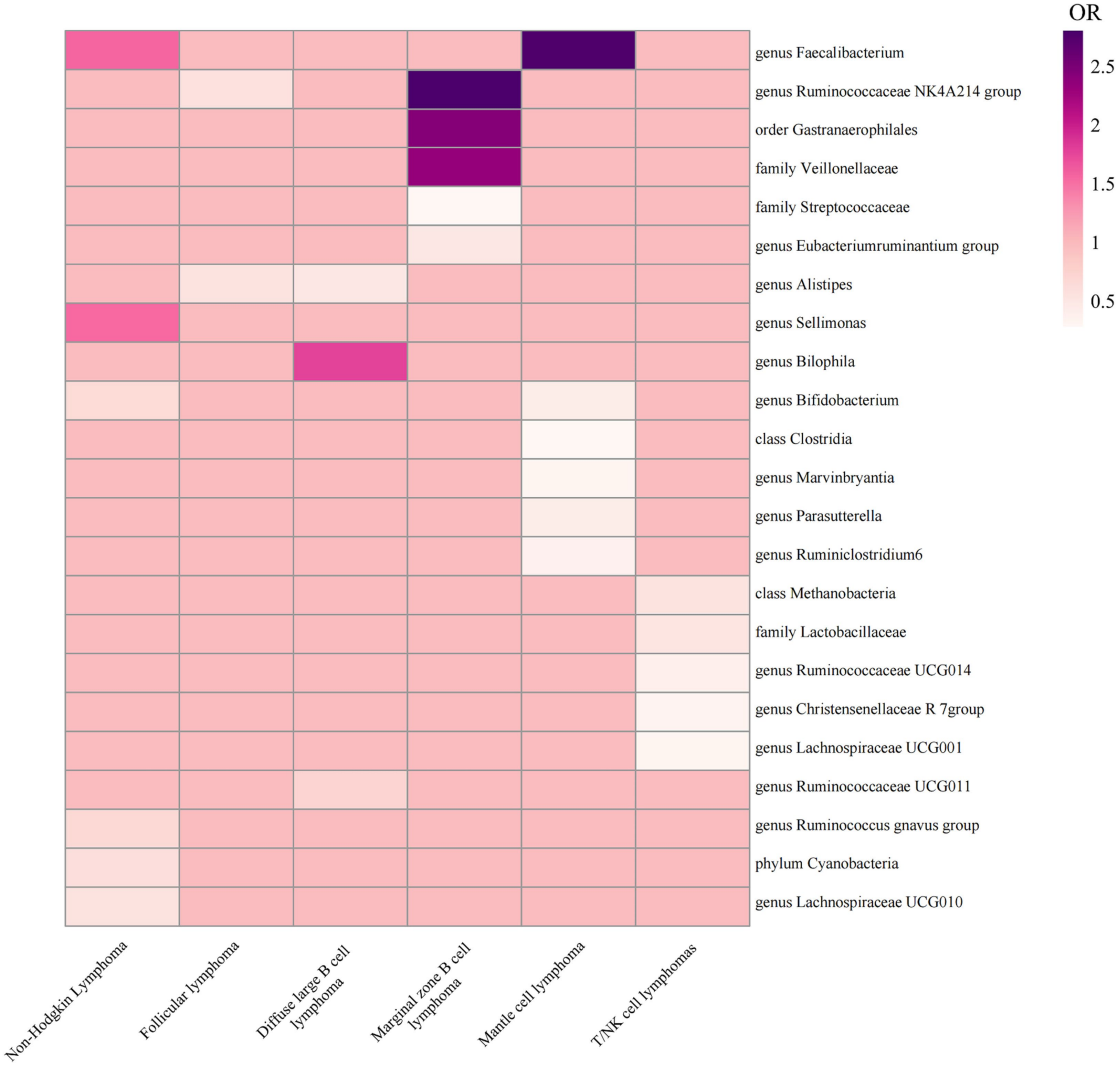
Heatmap of ORs in MR results for 27 GMs and NHL.

**Figure 6:**
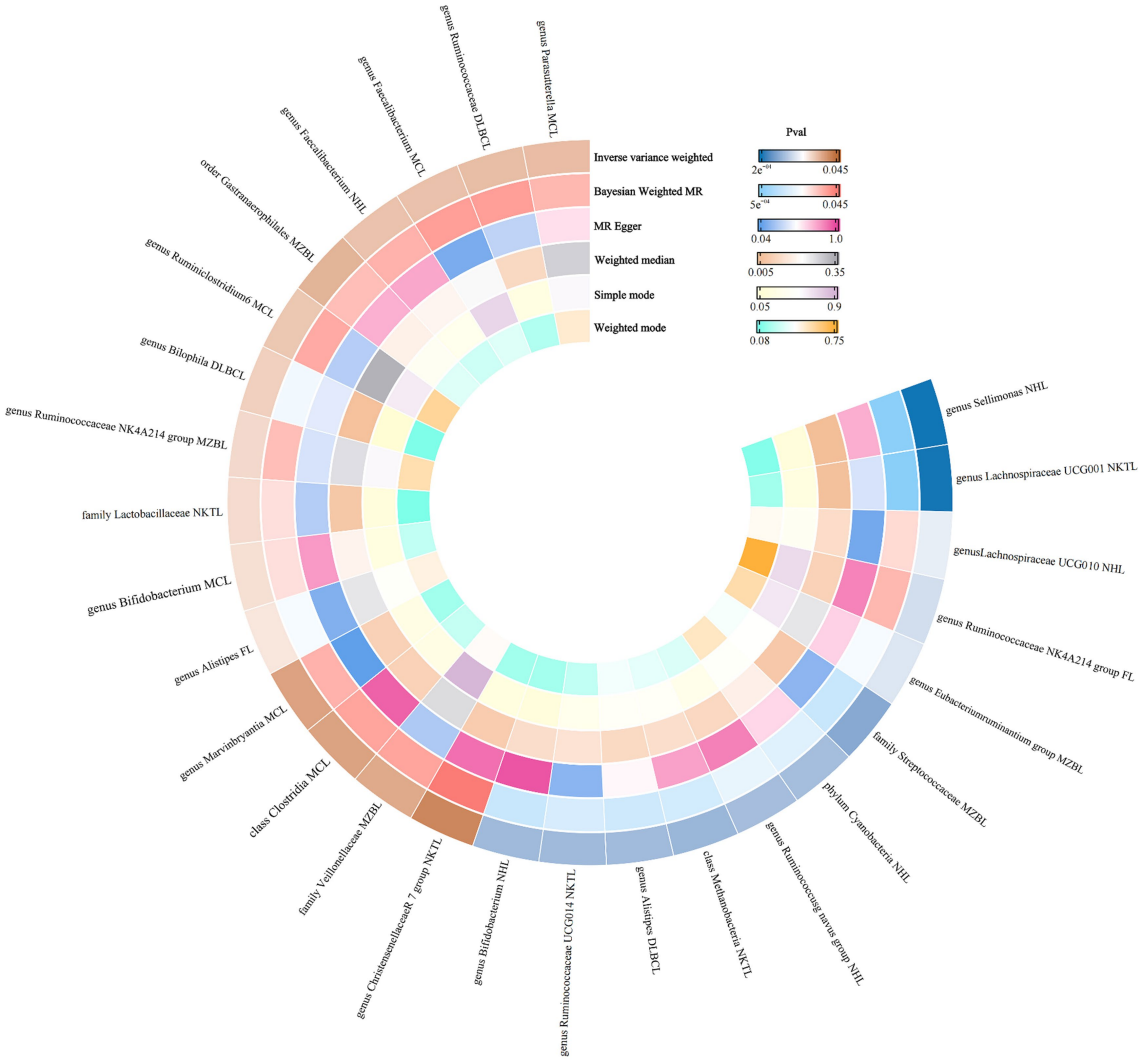
Ring thermograms of different MR results for 27 GMs and NHL.

Therefore, we finally identified 27 GMs with clear causal relationships with NHL and its subtypes, and presented them in the form of a heatmap (Figures 5, 6).

## 4 Discussion

In the 2020 cancer diagnosis statistics, NHL ranked 11th, and its incidence has been increasing year by year (Sung et al., 2021). Although GMs play important roles in the occurrence, development, diagnosis, and treatment of NHL (Upadhyay Banskota et al., 2023), the specific causality between the two is unknown. Previous studies have investigated the causal relationship between lipids (Kleinstern et al., 2020) and diet (Zhou et al., 2024), among other factors (Shi et al., 2024), and NHL through MR. Our research identified 27 GMs with causal relationships with NHL and its subtypes through forward and reverse MR analyses, as well as sensitivity analysis. Among them, phylum Cyanobacteria, genus *Ruminococcus gnavus* group, genus *Bifidobacterium*, genus Lachnospiraceae UCG010, genus *Alistipes*, genus Ruminococcaceae NK4A214 group, genus Ruminococcaceae UCG011, family Streptococcaceae, genus *Eubacterium ruminantium* group, class Clostridia, genus *Marvinbryantia*, genus *Parasutterella*, genus *Ruminiclostridium* 6, class Methanobacteria, family Lactobacillaceae, genus Christensenellaceae R 7group, genus Lachnospiraceae UCG001, and genus Ruminococcaceae UCG014 were negatively associated with the disease (OR < 1), indicating a protective effect against the corresponding types of NHL. Genus *Faecalibacterium*, genus *Sellimonas*, genus *Bilophila*, order Gastranaerophilales, family Veillonellaceae, genus Ruminococcaceae NK4A214 group, and genus *Faecalibacterium* were positively associated with the disease (OR > 1), serving as risk factors for the corresponding types of NHL. It is worth mentioning that in our positive MR analysis between GM and NHL, we observed that the absence of order Pasteurellales and genus *Haemophilus* might play a promoting role in FL occurrence. However, in the reverse MR analysis, we found that the occurrence of FL could inhibit the production of order Pasteurellales and genus *Haemophilus*. Therefore, we cannot ascertain whether the lack of order Pasteurellales and genus *Haemophilus* is the cause or the consequence of FL occurrence. To avoid interference from reverse causal relationships, we excluded the portion of results that exhibited reverse causal associations from the positive MR results.

The relationship between GM and NHL is complex. With the development of technologies in fields like 16S rRNA sequencing or shotgun metagenomics sequencing, researchers have gained a clearer understanding of the specific taxonomic groups in the GM and their relationship with diseases. Due to the involvement of numerous GM species in NHL and its subtypes in this study, we focused our discussion on the MR results related to NHL.

The phylum Cyanobacteria is a group of ancient and diverse prokaryotes (Schirrmeister et al., 2011) that can be divided into different genera such as Aphanothece, Leptolyngbya, and Spirulina (Walter et al., 2017). Research has found that Cyanobacteria can synthesize 1,600 types of compounds (Bohlin et al., 2010; Nagarajan et al., 2012), which play positive roles in antiviral, antibacterial, and immunomodulatory aspects (Sieber and Marahiel, 2005; De Morais et al., 2015; Sathasivam et al., 2019), thus they are widely applied in various fields. In addition, Cyanobacteria have significant anti-cancer effects, for example, Somocystinamide A (ScA), a lipopeptide compound isolated from Cyanobacteria, can inhibit tumor cell proliferation by inducing programmed cell death (Wrasidlo et al., 2008, p. 8). Curacin A, produced by Cyanobacteria, is a hybrid polyketide-peptide compound and an effective anticancer agent (Catassi et al., 2006). It can induce cancer cell death by inhibiting the activity of microtubule protein polymerization (Blokhin et al., 1995). In the results of this study, the phylum Cyanobacteria was found to decrease the risk of NHL, which is similar to the aforementioned previous research findings. Based on previous studies, we speculate that Cyanobacteria may also exhibit similar anticancer effects in NHL. Furthermore, most cancer-related chemotherapy drugs are derived from natural products in nature (Sithranga Boopathy and Kathiresan, 2010), and Cyanobacteria not only exist in the human gut but can also be obtained from marine (Mondal et al., 2020), soil, and agricultural runoff (Senousy et al., 2020). Hence, future research could delve into the anticancer mechanisms of Cyanobacteria in NHL, thereby laying the groundwork for the extraction and development of novel drugs related to NHL from natural products.

*Ruminococcus gnavus* is a Gram-positive anaerobic bacterium found primarily within the gastrointestinal tract of humans and animals (Qin et al., 2010). In terms of human health, *Ruminococcus gnavus* constitutes a significant proportion of the infant GM (Sagheddu et al., 2016), correlating with infant nutrition absorption (Yatsunenko et al., 2012) and growth development (Mennella et al., 2022), with these effects persisting into adulthood. In terms of disease, *Ruminococcus gnavus* is closely associated with gastrointestinal diseases and immune regulation. Research revealed that the relative abundance of *Ruminococcus gnavus* in normal humans is usually below 1%, while in some inflammatory bowel disease patients, it can reach around 70% (Zhang et al., 2023). However, some researchers found that after transferring *Ruminococcus gnavus* and other microbes into colorectal cancer mice, *Ruminococcus gnavus* could inhibit tumor growth and activate the immune surveillance function of CD8^+^ T cells (Zhang et al., 2023). Therefore, the role of *Ruminococcus gnavus* in the human body is complex, and its effects on disease are influenced by multiple factors. In this study, the genus *Ruminococcus gnavus* group was identified as a beneficial bacterium for NHL, which can reduce the risk of its occurrence. Lachnospiraceae and *Ruminococcus gnavus* belong to the phylum Firmicutes. Lachnospiraceae is a family of gut bacteria that is widely present in the gastrointestinal tract of fauna (Gosalbes et al., 2011; Meehan and Beiko, 2014). It is an important member of the human GM, accounting for approximately 10 to 45% of the total bacterial population (Liu et al., 2021). Lachnospiraceae can be divided into different genera, such as Lachnospira, Oribacterium, and Dorea (Vacca et al., 2020), which are the primary contributors to short-chain fatty acids that are beneficial to human health (Vital et al., 2014; Chambers et al., 2015; Bui et al., 2021). Meloxicam, a nonsteroidal anti-inflammatory drug, is associated with reducing the risk of cancer, while Lachnospiraceae can produce meloxicam by altering the heterocyclic structure of flavonoids (Sugiyama et al., 2013; Braune and Blaut, 2016). Although there is limited research on the association between Lachnospiraceae and NHL, and it is not commonly found in other hematological tumors (Guevara-Ramírez et al., 2023), the viewpoint of Lachnospiraceae being considered beneficial bacteria in previous literature is similar to the results of this study. Therefore, more attention should be paid to the study of the association between the phylum Firmicutes and NHL as well as blood tumors, to clearly elucidate the specific mechanisms by which Lachnospiraceae may contribute to the treatment of NHL, thereby providing new insights into the prevention and treatment of NHL.

Bifidobacterium is a well-recognized beneficial microorganism for human health (Hidalgo-Cantabrana et al., 2017; Leser and Baker, 2023), with functions such as inhibiting intestinal pathogens (Moreno Muñoz et al., 2011), preventing gastrointestinal infections (Weizman et al., 2005), improving gastrointestinal symptoms (Waller et al., 2011), and regulating the immune system (Roller et al., 2007), thus it is widely used in the food and pharmaceutical industries. Studies have shown that GM such as Bifidobacterium can influence the therapeutic effects of immunotherapy on tumors (Matson et al., 2018). Bifidobacterium can also enhance the efficacy of ICIs in cancer mice by producing adenosine (Mager et al., 2020). Chimeric antigen receptor T-cell immunotherapy (CAR-T) is a novel precision-targeted therapy for treating malignant tumors of the hematopoietic system. Studies have found that GM such as Bifidobacterium are associated with the efficacy of Chimeric antigen receptor T-cell therapy in BCL and can influence the progression of BCL (Stein-Thoeringer et al., 2023). In addition, researchers have observed that the diversity of Bifidobacterium in multiple myeloma people after receiving CAR-T therapy vary depending on the efficacy of the treatment (Hu et al., 2022). Therefore, the differences in the diversity and abundance of Bifidobacterium are important indicators for predicting the therapeutic effects of lymphoma and other malignant tumors of the hematopoietic system. This study found that Bifidobacterium can effectively reduce the risk of NHL, while previous literature has not addressed this aspect of research. Therefore, we hope that this study can provide valuable reference for future exploration. Moreover, existing studies indicate an association between Bifidobacterium and the immunotherapeutic effects on tumors such as BCL and multiple myeloma, yet they do not directly establish a connection between Bifidobacterium and BCL. Therefore, subsequent research can use this as a starting point.

In this study, we found that genus *Faecalibacterium* and genus *Sellimonas* are the only two intestinal microbiota that can increase the risk of NHL. However, it is worth noting that genus *Faecalibacterium* is commonly found in the population and is generally considered beneficial to health, with the potential to become the next generation of probiotics (Langella et al., 2019). For example, *Faecalibacterium prausnitzii*, an important member of the genus *Faecalibacterium*, constitutes more than 5 percent of the overall fecal microbiome of healthy individuals. It can maintain the stability of the healthy gut environment (Miquel et al., 2013) and also act as a probiotic to regulate the intestinal environment of Crohn’s disease patients (Sokol et al., 2008). Additionally, some species within the genus *Faecalibacterium* can produce significant amounts of fructose, providing energy for human colonic epithelial cells and supporting epithelial cell growth (Fagundes et al., 2021; Park J.-H. et al., 2022). Therefore, the results regarding genus *Faecalibacterium* in this study differ somewhat from previous related research. However, some researchers suggest that the interaction between *Faecalibacterium* and its host is not always constant (Martín et al., 2023). Since the discovery of *Faecalibacterium*, with the continuous advancement of techniques such as 16S rRNA gene sequence as well as whole-genome sequencing, the taxonomy of this genus has been evolving. In 2021, two new species were added: *Faecalibacterium butyricigenerans* and *Faecalibacterium longum* (Zou et al., 2021); and in 2022, three more new species were discovered, namely: *Faecalibacterium duncaniae*, *Faecalibacterium hattorii*, and *Faecalibacterium gallinarum* (Sakamoto et al., 2022). Therefore, the interactions between the genus *Faecalibacterium* and the host are continually being updated. Further research is needed to explore the impact of *Faecalibacterium* on NHL.

The causal relationship between GM and NHL is influenced not only by internal factors but also by external factors such as diet, medication, and delivery type. Dietary fiber is an important nutrient that is difficult for the human body to digest and absorb. However, there is a significant association between a high consumption of fruits, soy, and green vegetables and a reduced risk of NHL (Chiu, 1996; Wei et al., 2016). It is worth noting that certain GM, such as Lachnospiraceae, can ferment dietary fiber and produce substances like short-chain fatty acids, increasing the content of butyrate in the body, thereby promoting apoptosis of lymphoma cells (Wei et al., 2016; Zaplana et al., 2024). Therefore, increasing the intake of dietary fiber in the body appropriately can promote the growth of GM such as Lachnospiraceae. Additionally, certain living biotherapeutic products (LBPs) associated with Lachnospiraceae have been attempted to be developed as probiotics to improve conditions such as metabolic syndrome (Gilijamse et al., 2020). Probiotics are a type of beneficial active microorganisms for the human body. Bifidobacterium, as a crucial member of probiotics, plays an important role in the prevention and treatment of cancers such as colon cancer (Bahmani et al., 2019), gastric cancer (Devi et al., 2021), breast cancer (Shimizu et al., 2020), and lung cancer (An et al., 2020). This study identified a significant number of GM, including Bifidobacterium and *Ruminococcus gnavus*, that may potentially reduce the risk of NHL. Whether these GM can participate in the prevention and treatment of NHL as probiotics or other forms such as LBPs in the future is worth exploring.

The research has a few restrictions. Firstly, since the data on GM and NHL and its subtypes are all from European populations, we cannot guarantee whether the results are applicable to other populations. The GM dataset included in this study is currently the largest GWAS dataset of GM, but it predominantly focuses on the European population, hence there are limitations in generalizing to other populations. As GWAS databases of GM in various populations continue to be updated, we will continue to monitor research on the causal relationship between GM and NHL in other populations. Secondly, there are fewer cases in certain subtypes of NHL, such as MZBL, MCL, and T/NK cell lymphoma, which limits the scope of the study. We will continue to monitor this aspect of the research as the FinnGen database is continually updated. Lastly, this study only elucidates the causality between GM and NHL, and the underlying mechanisms driving this association are not yet clear, requiring further research for support.

## 5 Conclusion

Through this study, we have identified the causality between GM and NHL, and determined the beneficial and harmful microbiota for NHL. In the future, it may be considered to selectively alter these GM through measures such as diet, probiotics, and prebiotics to influence NHL. Additionally, the development of targeted and effective GM in clinical settings holds certain reference significance as novel therapeutic modalities and monitoring indicators for NHL. Therefore, this research offers novel ideas to prevent, monitor, and cure NHL later.

## Data availability statement

The original contributions presented in the study are included in the article/Supplementary material, further inquiries can be directed to the corresponding author.

## Author contributions

JF: Data curation, Writing – original draft, Writing – review & editing, Software. ZH: Funding acquisition, Project administration, Resources, Supervision, Validation, Writing – review & editing.
