# Supplementary material for: The causality between gut microbiota and non-Hodgkin lymphoma: a two-sample bidirectional Mendelian randomization study
Source: Front Microbiol. 2024 May 27;15:1403825. doi: 10.3389/fmicb.2024.1403825 (PMC11163074; doi:10.3389/fmicb.2024.1403825)
Supplement: Supplementary file 4 [file Data_Sheet_1.docx]

Supplementary Material

## Supplementary Figures


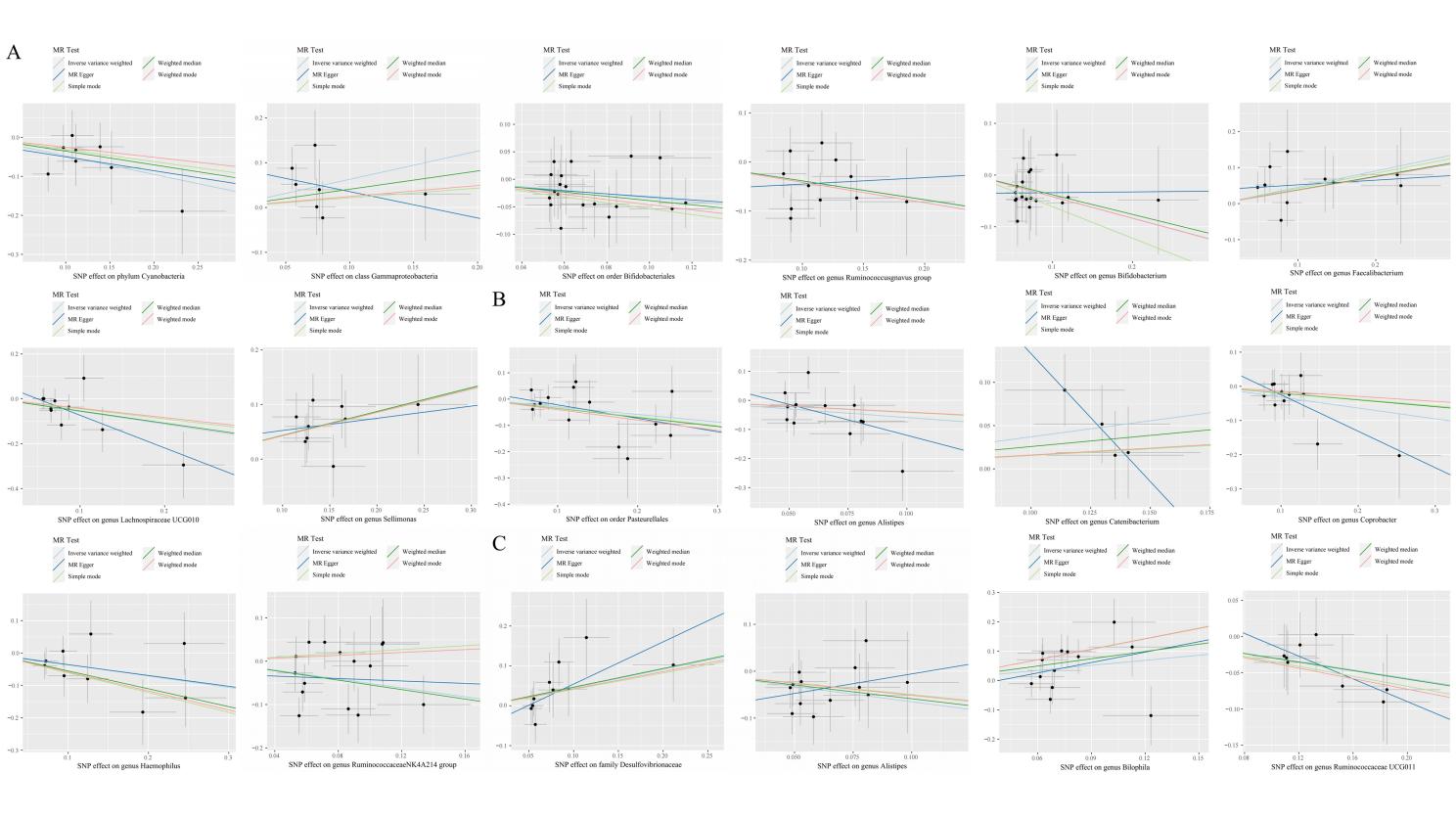


**Supplementary Figure 1.** Scatterplot of GM and NHL in the presence of causality: **(A)** GM and NHL; **(B)** GM and FL; **(C)** GM and DLBCL.

**
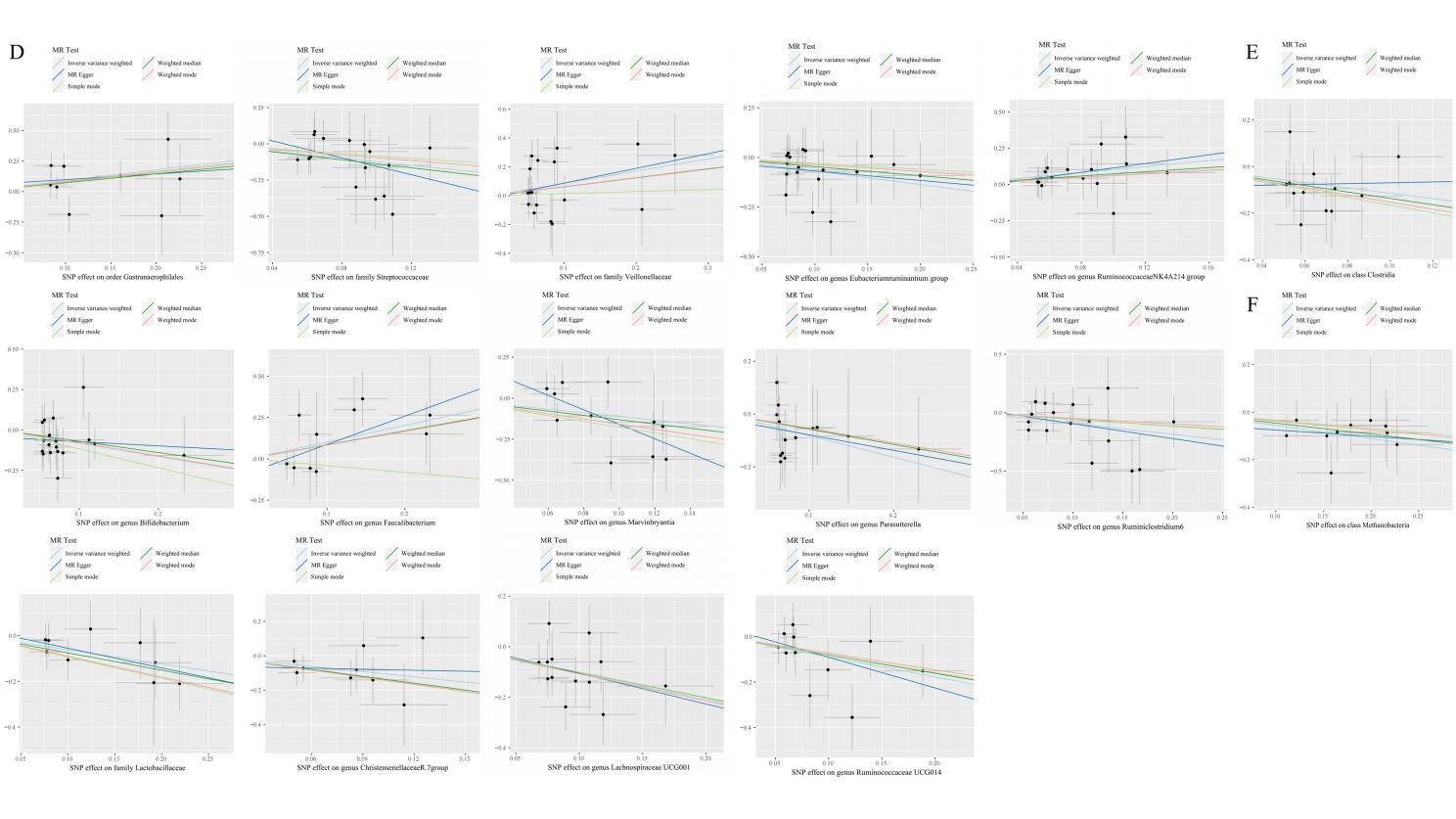
**

**Supplementary Figure 2.** Scatterplot of GM and NHL in the presence of causality: **(D)** GM and MZBL; **(E)** GM and MCL; **(F)** GM and NK/T cell lymphoma.


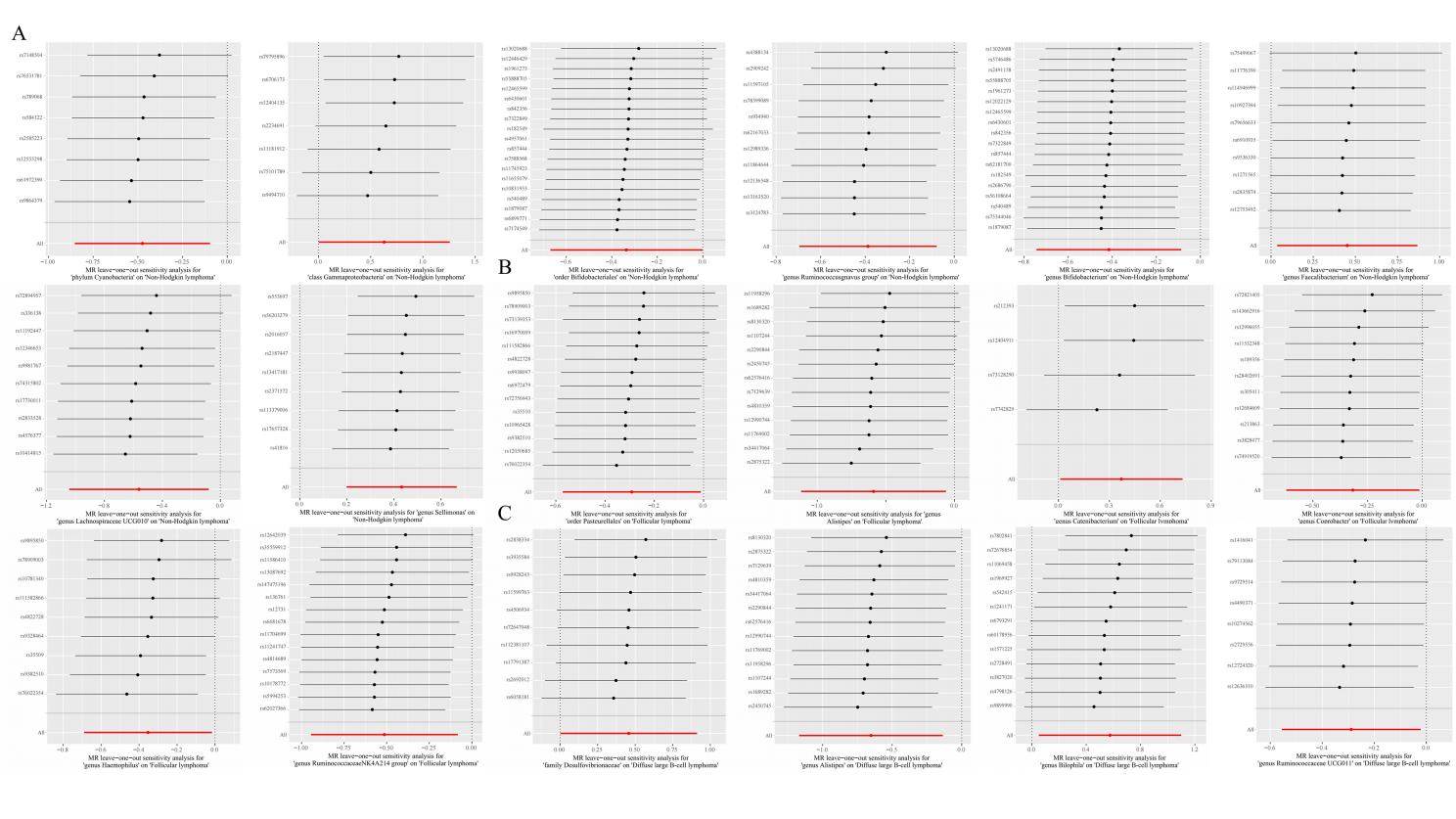


**Supplementary Figure 3.**  Forest plot of leave-one-out sensitivity analysis for GM and NHL in the presence of a causal relationship: **(A)** GM and NHL; **(B)** GM and FL; **(C)** GM and DLBCL.

**
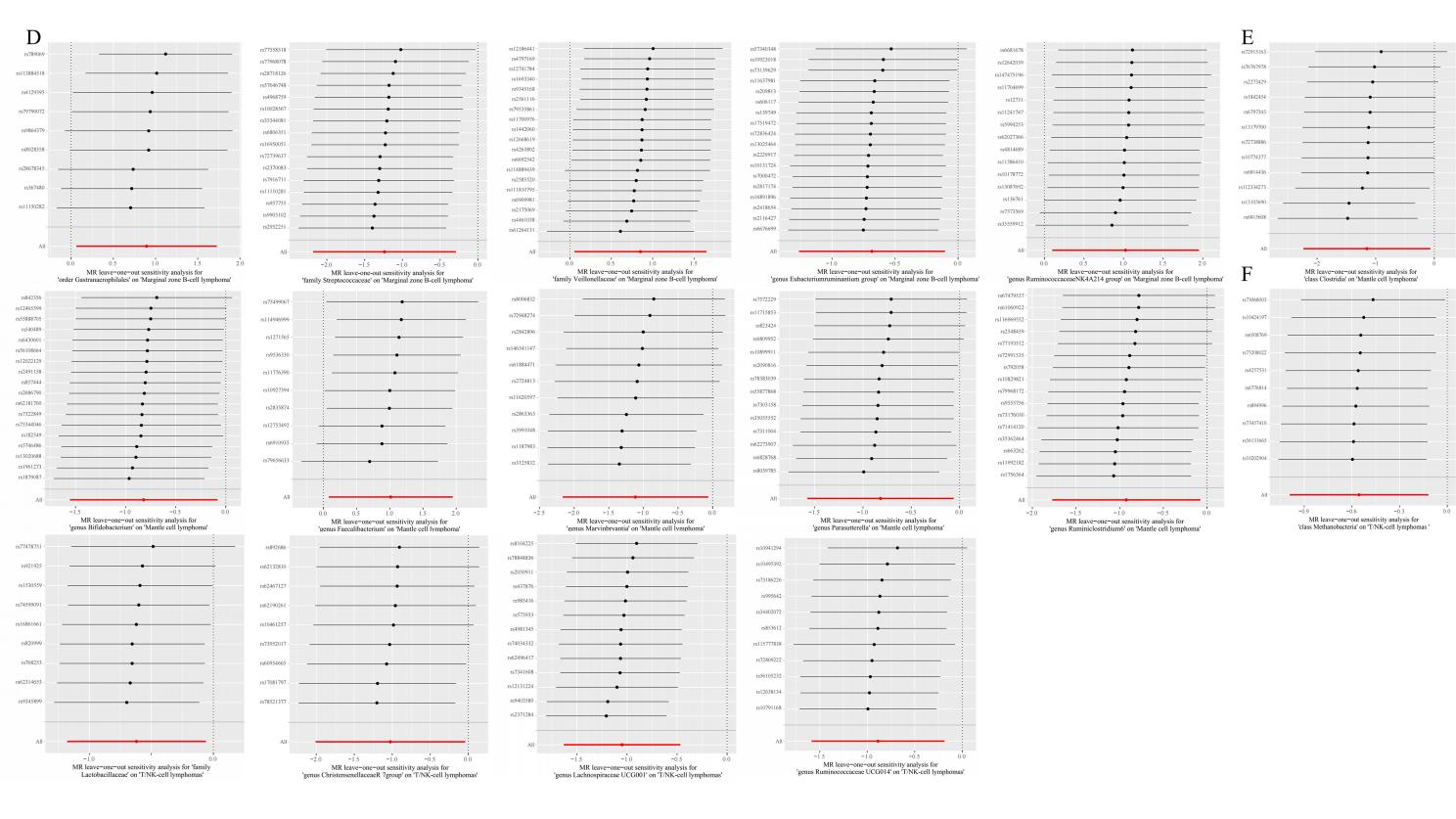
**

**Supplementary Figure 4.** Forest plot of leave-one-out sensitivity analysis for GM and NHL in the presence of a causal relationship: **(D)** GM and MZBL; **(E)** GM and MCL; **(F)** GM and NK/T cell lymphoma.

## Supplementary Tables

**Supplementary Table 1.** Detailed information of IVs used in MR analyses.

**Supplementary Table 2.** GM and NHL positive MR results and sensitivity analysis results.

**Supplementary Table 3.** Inverse MR results for GM and NHL.
